# Effectiveness Of Pre-operative Oral Medication of Ibuprofen and Ketorolac on Anesthetic Efficacy of Inferior Alveolar Nerve Block with Irreversible Pulpitis: Randomized Controlled Trial

**DOI:** 10.7759/cureus.6346

**Published:** 2019-12-11

**Authors:** Sainath Reddy Kaladi, Veeresh Tegginmani, Manoosha M, Sumana Mitta, Pradeep Chigadani, Anitha Viswanadhan

**Affiliations:** 1 Conservative Dentistry and Endodontics, MNR Dental College and Hospital, Hyderabad, IND; 2 Conservative Dentistry & Endodontics, AME's Dental College and Hospital, Raichur, IND; 3 Prosthodontics, Clove Dental Hospital, Hyderabad, IND; 4 Conservative Dentistry & Endodontics, AME's Dental College & Hospital, Raichur, IND; 5 Conservative Dentistry & Endodontics, Vishnu Dental College, Bhimavaram, IND

**Keywords:** nsaid’s, pre- operative pain, verbal rating scale, ianb

## Abstract

Background and objectives

Pain is the primary reason that dental patients seek endodontic therapy. The inferior alveolar nerve block (IANB) is the most frequently used mandibular injection technique for achieving local anesthesia for endodontic treatment. However, the IANB does not always result in successful pulpal anesthesia. Therefore, the purpose of this study was to determine the effects of preoperative administration of both ibuprofen and ketorolac on the efficacy of the IANB in patients with irreversible pulpitis.

Methods

A total of 60 patients diagnosed with irreversible pulpits of a mandibular posterior tooth randomly received identical capsules of either 400 mg ibuprofen or 20 mg ketorolac or a placebo 1 hour before the administration of a conventional IANB. Access was initiated after profound lip numbness was achieved. Success was defined as no, mild, moderate, or severe pain (verbal rating scale recordings) on accessing the dentin, pulp, and debridement.

Results

Ketorolac was associated with superior efficacy in pain reduction when compared with ibuprofen and placebo in all parameters, namely the dentin, pulp, and canal debridement.

Interpretation and conclusion

In conclusion, for mandibular posterior teeth, a preoperative dose of 400 mg of ibuprofen or 20 mg of ketorolac showed a statistically significant increase in the success of the IANB in patients with irreversible pulpitis.

## Introduction

For the endodontic treatment to be considered successful and accepted readily by the patient and dentist, it must efficiently relieve pain. Local anesthesia, through inhibition of nociceptive signals, is integral to the management of painful endodontic emergencies [1]. A study found that a single inferior alveolar nerve block (IANB) with lignocaine is ineffective in 30% to 80% of the patients diagnosed with irreversible pulpitis [2]. Clinical studies in endodontics have found failure with IANB in 15% of patients with normal tissue and 44% to 81% patients with irreversible pulpitis [3-4]. Various mechanisms are cited to explain the failure of local anesthetics. The mechanisms include decreased local pH and activation of nociceptors, such as tetrodotoxin and capsaicin-sensitive transient receptor potential vanilloid type 1.

Prostaglandin-induced sensitization of peripheral nociceptors has been commonly implicated in the high failure rate of local anesthesia in symptomatic teeth with irreversible pulpitis, which is caused by inflammation [5-6]. Non-steroidal anti-inflammatory drugs (NSAIDs) reduce nociceptor activation by decreasing the levels of inflammatory mediators. Premedication with NSAIDs can improve the success rate of local anesthesia in patients with irreversible pulpitis [3].

The aim of this study is to compare the effectiveness of pre-operative oral medication with ibuprofen and ketorolac on anesthetic efficacy in an IANB with 1:80000 epinephrine in patients with irreversible pulpitis.

## Materials and methods

This was a randomized controlled trial. The study plan was approved by the institutional ethical committee (Ethical certificate no: AME/DC/IEC/MD-9/2015-16). Using n - Master Sample Size Calculation Software with 88% power and 95% confidence interval, the sample size was found to be 60, that is, *n* = 20 per each group. Patients with symptomatic irreversible pulpitis of any molar teeth that responded to cold testing with an ice stick and an electric pulp tester without periapical radiolucency on a radiograph, except for widened periodontal ligament, with good systemic health were included. Patients who had NSAIDs within 12 hours of administration of the study drugs, pregnant and breastfeeding patients, and those with a history of active peptic ulcer or bleeding or anticoagulant use within the last month were excluded. The participants received information regarding the nature of the study. Written consent was obtained from each participant.

Prior to injection, the experimental tooth and the contralateral tooth were tested with a Digitest electric pulp tester (Parkell, Farmingdale, NY, USA) to obtain a baseline score. Subsequently, patients with prolonged response to the cold test were selected. The study patients were grouped into three categories based on oral analgesics as follows:

Group 1 (*n *= 20): without premedication

Group 2 (*n *= 20): with premedication of ibuprofen tablet

Group 3 (*n *= 20): with premedication of ketorolac tablet

After one hour of oral study drug administration, lidocaine topical anesthesia was sprayed at the region of injection followed by IANB with 2% lidocaine with 1:80,000 epinephrine under sterile conditions. The depth of anesthesia was monitored based on lip numbness, after 15 minutes of the initial IANB. If profound lip numbness was not recorded within 15 minutes, the block was considered unsuccessful, and the patient was excluded from the study. The involved teeth were isolated with a rubber dam, and a conventional access opening was initiated. In case of pain during treatment, the procedure was stopped and the patient was required to rate the pain using a verbal rating scale (VRS). The treatment was divided into three phases: access into dentin, access into the pulp chamber, and canal instrumentation. Using a VRS, the extent of pain was recorded within the dentin and pulpal space and during canal instrumentation.

## Results

The data were subjected to descriptive statistics and, non-parametric tests by using SPSS software, version 23 (IBM Corporation, Armonk, New York). Table 1 shows the mean age of the study population according to groups and gender. In the ketorolac group, the mean age group of males (13/20) was 31.46 ± 4.156, and the mean age group of females (7/20) was 33.71 ± 3.147. In the ibuprofen group, the mean age group of males (11/20) was 32.55 ± 3.671, and the mean age group of females (9/20) was 29.22 ± 4.969. In the placebo group, the mean age group of males (13/20) was 29.92 ± 4.974, and the mean age group of females (7/20) was 32.29 ± 3.450.

**Table 1:** Mean age of study population according to group and gender

Group	Gender	N	Minimum	Maximum	Mean	Standard Deviation
Ketorolac Group	Male	13	24	38	31.46	4.156
	Female	7	29	39	33.71	3.147
Ibuprofen group	Male	11	27	37	32.55	3.671
	Female	9	24	36	29.22	4.969
Placebo Group	Male	13	24	39	29.92	4.974
	Female	7	27	37	32.29	3.450

The frequency distribution and median of dentin pain based on VRS scores among various groups calculated using chi-square test were highly statistically significant (*P *< 0.001; Table 2).

**Table 2:** Frequency distribution of dentin VRS score VRS, verbal rating scale; **Highly significant (*p *< 0.01)

Score	Ketorolac Group		Ibuprofen Group		Placebo Group	
	Frequency	Percentage	Frequency	Percentage	Frequency	Percentage
1	0	0	0	0	0	0
2	16	80.0	0	0	0	0
3	4	20.0	16	80.0	10	50.0
4	0	0.0	4	20.0	10	50.0
Total	20	100.0	20	100.0	20	100.0
Median	2		3		4	
Chi-square value	50.05		P value		<0.001**	

The frequency distribution and median of pulp pain VRS scores among various groups were statistically significant (Table 3).

**Table 3:** Frequency distribution of pulp VRS score VRS, verbal rating scale; *Statistically significant (*p *< 0.05)

Score	Ketorolac Group		Ibuprofen Group		Placebo Group	
	Frequency	Percentage	Frequency	Percentage	Frequency	Percentage
1	0	0	0	0	0	0
2	2	10.0	0	0	0	0
3	10	50.0	11	55.0	4	20.0
4	8	40.0	9	45.0	16	80.0
Total	20	100.0	20	100.0	20	100.0
Median	3		3		4	
Chi sq	10.895		P-value		0.028*	

The frequency distribution and median of debridement pain VRS scores between various groups were highly significant (*P *< 0.01), calculated using the chi-square test (Table 4).

**Table 4:** Frequency distribution of debridement VRS score VRS, verbal rating scale; **Highly significant (*p *< 0.01)

Score	Ketorolac Group		Ibuprofen Group		Placebo Group	
	Frequency	Percentage	Frequency	Percentage	Frequency	Percentage
1	19	95.0	9	45.0	0	0
2	1	5.0	11	55.0	3	15.0
3	0	0.0	0	0.0	17	85.0
4	0	0.0	0	0.0	0	0.0
Total	20	100.0	20	100.0	20	100.0
Median	1		2		3	
Chi sq	64.557		P-value	<0.001**		

Intragroup comparison was performed using Friedman test (horizontal H value) to assess the significant difference in pain scores at various locations in each group. For individual comparison, Wilcoxon signed-rank test was performed. Median pain scores at baseline versus the dentin group showed statistically significant efficacy of ketorolac (*P* < 0.001) compared with that of ibuprofen (*P* < 0.012); the efficacy of the placebo was not statistically significant compared with the baseline (*P* > 0.132). In baseline versus pulp extirpation, the efficacy of ketorolac (*P* < 0.016) and ibuprofen (*P* < 0.025) was statistically significant and that of placebo (0.739) was statistically non-significant. In baseline versus canal debridement, all three median pain scores showed high significance; ketorolac: *P* < 0.001, ibuprofen: *P* < 0.001, placebo: *P* < 0.001. In dentin removal versus pulp extirpation, the ketorolac (*P* < 0.001) group was highly significant compared with the placebo (0.034), and ketorolac and the placebo were statistically significant and statistically nonsignificant, respectively, compared with ibuprofen (0.166). In dentin removal versus canal debridement, all three groups showed high significance; ketorolac: (*P* < 0.001), ibuprofen: (*P* < 0.001), and placebo: (*P* < 0.002). In pulp versus canal debridement, all three groups showed high significance; ketorolac: *P* < 0.001, ibuprofen: *P* < 0.001, and placebo: *P* < 0.001 (Table 5).

**Table 5:** Intragroup comparison of pain scores at various locations **Statistically significant (*p *< 0.01), *Statistically significant (*p *< 0.05); NS, not significant (*p* > 0.05)

Comparison	Ketorolac group		Ibuprofen group		Placebo group	
	Z value	Sig	Z value	Sig	Z value	Sig
Baseline vs Dentin	-4.072	<0.001**	-2.5	0.012*	-1.508	0.132 NS
Baseline vs Pulp	-2.399	0.016*	-2.236	0.025*	-0.333	0.739 NS
Baseline vs Debridement	-4.179	<0.001**	-3.976	<0.001**	-4.243	<0.001**
Dentin vs Pulp	-3.704	<0.001**	-1.387	0.166 NS	-2.121	0.034*
Dentin vs Debridement	-4.065	<0.001**	-4.028	<0.001**	-3.127	0.002**
Pulp vs Debridement	-3.993	<0.001**	-3.976	<0.001**	-3.755	<0.001**

Intergroup comparison of the visual analogue scale (VAS) score was done performed using Kruskal-Wallis test (vertical H value), whereas Mann-Whitney U test was performed for individual pairwise comparisons. At every location, the ketorolac group showed low pain scores, whereas the placebo group showed the highest pain scores. Therefore, the ketorolac group presented the highest premedication pain reduction compared with the ibuprofen and placebo groups.

The ketorolac group showed a baseline score of mean rank 33.00 and median of 4. In the dentin, the mean rank was 13.10 and median was 2(50%); in the pulp, the mean rank was 25.25 and median was 3(25%); and in the debridement, the mean rank was 15.58 and median 1(75%), which was highly significant (*P* < 0.001) as per Friedman value 54.326 (Table 6).

**Table 6:** Intergroup comparison of VAS scores VAS, visual analogue scale; **Statistically highly significant (*p *< 0.01); NS, not significant (*p *> 0.05)

	Intragroup Comparison											
Intergroup comparison	Group	N	Baseline		Dentin		Pulp		Debridement			
			Mean Rank	Median	Mean Rank	Median	Mean Rank	Median	Mean Rank	Median	Friedman value	Sig
	Ketorolac Group	20	33.00	4	13.10	2 (50%)	25.25	3 (25%)	15.58	1 (75%)	54.326	<0.001**
	Ibuprofen Group	20	28.50	4	35.90	3 (25%)	28.05	3 (25%)	26.33	2 (50%)	46.053	<0.001**
	Placebo Group	20	30.00	4	42.50	4 (0%)	38.20	4 (0%)	49.60	3 (25%)	31.867	<0.001**
	H value	1.283			42.45		32.97		45.75			
	Sig	0.527 NS			<0.001**		<0.001**		<0.001**			

Table 7 shows intergroup comparisons of pain at different locations. At dentin and debridement, there was a statistically significant difference between each group. At pulp, there was no statistically significant difference between ketorolac and ibuprofen groups, which indicated that both are equally efficacious in pain reduction during pulp extirpation.

**Table 7:** Intergroup comparison of pain scores **Statistically highly significant (*p *< 0.01); NS, not significant (*p *> 0.05)

Group	N	Baseline		Dentin		Pulp		Debridement	
		Z value	Sig	Z value	Sig	Z value	Sig	Z value	Sig
Ketorolac Group vs Ibuprofen Group	20	-1.122	0.429 NS	-5.048	<0.001**	-0.644	0.583 NS	-3.407	0.006**
Ketorolac group vs Placebo Group	20	-0.781	0.602 NS	-5.196	<0.001**	-2.639	0.023*	-5.946	<0.001**
Ibuprofen group vs Placebo group	20	-0.350	0.799 NS	-1.964	0.108 NS	-2.257	0.060 NS	-5.323	<0.001**

## Discussion

The International Association for the Study of Pain defines pain as “an unpleasant sensory or emotional experience associated with actual or potential tissue damage or described in terms of such damage”[7]. Pain is always subjective. Each individual learns the application of the word through experiences related to injury in early life [8]. The perceived perception of pain with endodontic therapy is a significant source of fear for many patients and can prevent them from seeking dental treatment. Therefore, a key objective of endodontic therapy is to relieve or prevent patients' pain during the treatment to control their anxiety and fear [8]. Hence, achieving adequate anesthesia in painful pulps is essential before we approach the pulp.

The conventional IANB technique is used for mandibular nerve block, as the technique requires minimal experience and the bony landmarks can be easily located. A study by Thangavelu et al. (2012) showed a success rate of 85% and 95% with conventional and Gow-Gates techniques, respectively [9]. The most commonly used anesthetic solution in dental practice, that is, 1:80000 epinephrine with 2% lignocaine was used, which is compatible with all vasoconstrictors, presents extremely rare chances of allergic reactions and exhibits rapid onset (2-3 minutes) [10]. Lidocaine is the “gold standard” drug with which all new local anesthetics are compared.

The precise level of vasoconstrictor in a local anesthetic solution necessary to provide sufficient pulpal anesthesia is unknown. The clinical application of a vasoconstrictor is to attain hemostasis, decrease absorption into the cardiovascular system, and increase the duration of anesthesia. The present study agrees with the findings by Dagher et al. (1997) where the different concentrations of epinephrine (1:50000, 1:80000, and 1:100000) with 2% lidocaine in the IANB showed no significant difference in the success rates [11].

Patients with irreversible pulpitis presented an eight-fold higher failure of local anesthetic injections than control patients [2,12]. Therefore, local anesthetic failures can occur in a substantial proportion of endodontic pain patients. Clinical studies have reported that a single IANB injection of lidocaine (1.8 cc) is ineffective in 30% to 80% of patients with a diagnosis of irreversible pulpitis [2,12]. This failure of IANB with lidocaine is associated with various factors, particularly in cases with previous inflammation [13].

Pulpal pain is a type of visceral deep somatic pain; therefore, it has a high threshold and poor localization. Several studies have shown that inflammation increases the interstitial fluid pressure in the dental pulp. The absolute magnitude of the pressure increase in the locally inflamed area varied considerably. Dendritic cells present in the pulp release interleukin-8, which serves as a powerful chemotactic agent for other inflammatory cells, including neutrophils, macrophages, and mast cells. Histamines released from mast cells are potent vasodilators that cause vasodilation of arterioles and leakage of venules, which further increases inflammatory infiltration in the pulp and their chemical mediators. This increase in the infiltrate has two main consequences, both of which are synergetic: increase in tissue pressure and increase in inflammatory infiltration.

Prostaglandins upregulate various mechanisms that may decrease the efficacy of local anesthetics. Voltage-gated sodium channels are the target of local anesthetics. More than nine subtypes of sodium channels exist. Nociceptors express a tetrodotoxin-resistant class of voltage-gated sodium channels that are relatively resistant to lidocaine and are sensitized by prostaglandins. The specific isoforms Nav1.8 and Nav1.9 play a key role in the generation and maintenance of inflammatory pain. Prostaglandins alter the kinetics of the channels’ activity resulting in increased depolarization, whereas the actual channels themselves are expressed at higher levels [14]. In a recent study, Wells et al., using immunoreactivity, found significantly increased levels of the Nav1.9 isoform in painful and inflamed teeth compared with asymptomatic dental pulp [15].

Therefore, ibuprofen and ketorolac were used in this study as they reduce the inflammatory action and treat mandibular molars with irreversible pulpitis. Before beginning the procedure, baseline scores are noted as per the VRS using an electric pulp tester for each tooth. No pain was scored as 1; mild pain of score 2 was not shown in any group. The ketorolac group showed a moderate pain score 3 (15%) and severe pain score 4 (85%). The ibuprofen group showed 30% with score 3 and 70% with score 4. Placebo showed 25% with score 3 and 75% with score 4. VRS was used in this study as it can be readily assessed, is less time-consuming, can be performed without paper and pen, simple to understand for patients, and provides a correlation that is more definitive than a distance mark in a VAS.

To re-confirm the vitality of the pulp, the cold test was performed using spray refrigerant (Endo Ice) on a cotton pellet applied to the facial cervical areas of the teeth to be tested. Response to cold should be relatively consistent among normal teeth in a given area. Typically, a normal response would be immediate, possibly sharp and intense, but brief in duration (below 10 seconds). Duration and relative intensity of response was the key to making a diagnosis. An abnormal response would include one that is severe relative to surrounding teeth, or one that lasts longer than 10 seconds. Subsiding of pulsing or throbbing after the initial response would also be considered abnormal, a sign of irreversible pulp damage.

The odds of an accurate positive cold response as an indicator of pulp vitality is 90% versus 83% with the heat test and 84% with the electric test [16]. Furthermore, cold spray is more reliable than ice [13]. In the present study, symptomatic pulpitis was defined as a painful response to the cold stimulus that lingers for several minutes after the stimulus is removed. Only patients who met this criterion were included in this study.

In this study, the ibuprofen group showed 50% pain reduction in canal debridement, 25% in both pulp and dentin, and 0% in the baseline. Ibuprofen is a propionic acid derivative of NSAIDs and an effective analgesic, anti-inflammatory, and antipyretic and inhibits the synthesis of prostaglandins in body tissues through inhibition of cyclooxygenase-1 and -2. Ibuprofen 400 mg was used in this study as the daily recommended dose is 1200 mg/day (14 mg/kg) as per the Food and Drug Administration and Nonprescription Drug Advisory Committee. The over-the-counter dosing regimen of 200-400 mg has been shown to provide effective analgesia and allow flexibility in dosing where necessary.

In contrast to the present study, Seymour and Ward compared 200, 400, and 600 mg ibuprofen and noted a trend toward improved relief with 600 mg compared with 400 mg. Nielsen et al. showed that 800 mg ibuprofen was superior to 400 mg in a study of laser-induced pain [17-18]. Oleson et al. evaluated the effect of preoperative 800 mg ibuprofen on the efficacy of the IANB in irreversible pulpitis, which showed a success rate of 41% with ibuprofen and 35% with placebo [19]. Seymour and Ward evaluated various doses of ibuprofen (200, 400, and 600 mg) for the management of postoperative dental pain and reported a trend of increased pain relief in patients taking 600-mg doses [17]. In the present study, the ketorolac group showed 75% pain reduction in debridement, 50% in dentin, 25% in pulp, and 0% in the baseline. Ketorolac is a pyrrolo-pyrrole derivative of NSAIDs. Most of the analgesic actions of NSAIDs are attributed to the inhibition of prostaglandin synthesis in the periphery and prevention of peripheral sensitization. Nociceptors express receptors for both PGE2 and PGI2 prostaglandin products of arachidonic acid metabolism, which is synthesized by the enzyme cyclo-oxygenase. It irreversibly acetylates enzyme cyclooxygenase and thereby blocks the production of prostaglandins. Prostaglandins sensitize nerve-endings to bradykinin, histamines, and other inflammatory mediators`; thus, they play a significant role in peripheral sensitization.

In a study by Vivek Aggarwal et.al. on irreversible pulpitis, premedication with IANB showed that ketorolac, ibuprofen, and placebo were associated with 39%, 27%, and 29% success rates, respectively [1]. Neighbor and Puntillo et.al. compared intramuscular ketorolac (50 mg) and oral ibuprofen (800 mg) for relief of acute pain and found them equivalent and effective in 60% of the cases [20]. The difference in pain reduction scores at dentin, pulp, and canal debridement may be due to myelination and smaller diameter of A-delta fibers, which result in a slower conduction velocity than other types of A-fibers; however, A-delta fibers are faster than C fibers. The A fibers transmit pain directly to the thalamus, generating a fast and, sharp pain that is easily localized. Pain in canals is due to the presence of C fibers in the intracanal region that are unmyelinated, larger in diameter, and are influenced by many modulating interneuron’s before reaching the thalamus, resulting in a slow pain, which is characterized as dull and aching [21]. The limitation of the present study is sample size. Further studies are also necessary to evaluate in a large population.

## Conclusions

Within the limitations of the study, it can be concluded that ketorolac is effective in the reduction of pre-operative pain in symptomatic teeth with IANB 1 hour prior to treatment. 
